# A Manual of Chemistry, for the Use of Medical Students

**Published:** 1890-01

**Authors:** 


**A Manual of Chemistry, for the Use of Medical Students.** By BRANDRETH SYMONDS, A. M., M. D., Assistant Physician to Roosevelt Hospital, Out-Patient Department; Attending Physician Northwestern Dispensary, New York. Pp. 154. Philadelphia: P. Blakiston, Son & Co. 1889.

Chemistry would seem to furnish a proper field for a well-prepared manual. It facilitates the acquirement of practical information by the student, while omitting much that can never be thoroughly acquired—much less applied—by any except those who expect to teach the science. It is our opinion, after a reasonably careful examination of Dr. Symonds's book, that it meets the claims of its author, and we think the student in search of such literature cannot fail to derive satisfaction from its possession.

